# Mechanisms of gait phase entrainment in healthy subjects during rhythmic electrical stimulation of the medial gastrocnemius

**DOI:** 10.1371/journal.pone.0241339

**Published:** 2020-10-23

**Authors:** Jenna E. Thorp, Peter Gabriel Adamczyk

**Affiliations:** Department of Mechanical Engineering, University of Wisconsin-Madison, Madison, Wisconsin, United States of America; Toronto Rehabilitation Institute - UHN, CANADA

## Abstract

Studies have shown that human gait entrains to rhythmic bursts of ankle torque for perturbation intervals both slightly shorter and slightly longer than the natural stride period while walking on a treadmill and during overground walking, with phase alignment such that the torque adds to ankle push-off. This study investigated whether human gait also entrains to align the phase of rhythmic electrical stimulation of the gastrocnemius muscle with the timing of ankle push-off. In addition, this study investigated the muscle response to electrical stimulation at different phases of the gait cycle. We found that for both treadmill and overground walking entrainment was observed with phasing that aligned the stimuli with ankle push-off or just before foot contact. Achilles tendon wave speed measurements showed a significant difference (increase) in tendon load when electrical stimulation was applied just after foot contact and during swing phase, with a greater increase for higher amplitudes of electrical stimulation. However, stimulation did not increase tendon load when the timing coincided with push-off. Stride period measurements also suggest the effect of electrical stimulation is sensitive to the gait phase it is applied. These results confirmed that timing aligned with push-off is an attractor for electrical stimulation-induced perturbations of the medial gastrocnemius, and that the muscle response to stimulation is sensitive to timing and amplitude. Future research should investigate other muscles and timings and separate sensory vs. motor contributions to these phenomena.

## Introduction

Locomotion is a common and important challenge for persons with neuromotor impairments. Management approaches vary depending on the condition, such as orthoses or robotic exoskeletons for stroke [1, 2] or spinal cord injury [3–5] and visual or auditory cueing for Parkinson’s disease [6, 7]. Most current solutions are highly obtrusive – physically large and/or noisy – making them suboptimal for practical use. For individuals who retain substantial natural movement capacity, many wearable solutions also interfere with this movement [8]. Therefore, much recent effort has aimed to develop therapeutic and assistive robotic devices that do not interfere with the natural dynamics of walking, such as soft exosuits [9–11] and backdrivable wearable robots [12–14]. Some efforts also aim to combine wearable systems with functional electrical stimulation neuroprostheses for a hybrid human-robotic system [15–17]. For these systems to be fully effective, it is important to understand the many ways in which the human and artificial systems interact.

One line of inquiry has studied how the body changes its movement in response to rhythmic mechanical perturbations. In one study, an exoskeletal ankle robot (Anklebot, Interactive Motion Technologies, Inc) was used to apply periodic plantarflexion torque pulses in parallel with the natural ankle in unimpaired persons walking on a treadmill. Using pulses of magnitude 10 N-m and duration 0.1 s (comparable to 10% of maximum ankle torque during normal walking for 10% of stride duration) at pulse periods ± 50 ms and ± 100 ms from their preferred cadence [13], this intervention caused 18 out of 19 subjects, in 42% of trials overall, to match their stride cadence to the perturbation period – a phenomenon called *entrainment* [13]. Subjects also converged to a common timing within the gait cycle, with the pulse phase-locked at 50.2 ± 3.8% of the gait cycle – coincident with the timing of push-off and maximum ankle actuation. A follow-up study showed similar results during overground walking [14, 18]. With perturbations only at ± 50 ms from the subjects’ preferred stride period, entrainment was observed in 82% of trials with a mean phase of convergence of 51.6 ± 2.4% [18]. Phase convergence occurred earlier and persisted longer in overground than in treadmill trials [14], perhaps because the constant speed imposed during treadmill walking conflicts with the strong natural coupling between speed and stride period [19, 20]. Overall, these results suggest mechanical perturbations at the ankle may be a feasible approach for walking rehabilitation. The assistive torque pulses could be used to improve gait by driving an individual to increase their cadence and walking speed.

However, the natural limb may not need a robot to apply such mechanical perturbations. The muscle system provides biological actuators at the ankle joint that are capable of producing very high torque. And, these muscles can be activated artificially through neuromuscular electrical stimulation (ES). Together these ideas suggest that rhythmic ES of the plantarflexors could be used to alter gait through entrainment. In this concept, an electrical stimulus would create a periodic biomechanical perturbation through the plantarflexor muscles, and the body would exploit this perturbation by matching its stride period to the stimulus period with the stimulus timed to the most beneficial phase of gait.

To our knowledge, there has only been one study testing whether entrainment can be obtained using electrical stimulation. This study investigated whether ES amplitude and stimulation site affect the level of entrainment while walking on a treadmill [21]. Perturbation periods of -60, -40, -20, +20, and +40 ms from the initial gait cycle periods were tested for stimulation sites on the hamstring and calf muscles, with two stimulation amplitudes: a full amplitude close to the limit each subject could tolerate in walking, and a reduced amplitude intended to cause a tingling sensation without ankle or knee motion. Entrainment was similar across the two stimulation sites and amplitudes (59% of subjects for the full amplitude stimulation and 51% with reduced amplitude) [21], but only occurred when the perturbation period was sufficiently close to the preferred stride period (± 20 ms perturbation period). This led them to conclude that entrainment was likely attributable to the sensory cues rather than electrically evoked motions that biomechanically assist gait. However, they did not report on whether the gait entrained to a specific phase of the gait cycle like in the previous ankle torque entrainment studies. They also only tested treadmill walking.

Viewed together, these prior studies suggest that electrical stimulation of the plantarflexor muscles could be an ideal tool for inducing changes in gait rhythm: electrical stimulation creates a direct sensory input, and the muscles could create a biomechanical input similar to that previously provided by the ankle robot. If successful, rhythmic electrical stimulation of the plantarflexors could lead to a new form of gait rehabilitation, for example to overcome gait disruptions in Parkinson’s disease or to promote rhythmic gait in stroke. A simple, noninvasive and unobtrusive electrode system would be a cost-effective way to rehabilitate gait or manage it as an assistive device. An example is the use of peroneal nerve stimulators for persons with foot-drop [22, 23]. However, further evidence of when and how electrical stimulation of the plantarflexors affects gait is necessary before such a clinical intervention can be developed.

This study builds on this prior research on rhythmic ankle stimuli to further investigate whether gait entrainment can be driven through the body’s own electromuscular response, and to what phase of the gait cycle entrainment phase-locking occurs. We hypothesized that rhythmic stimulation of the medial gastrocnemius would lead to entrainment as in the prior Anklebot and electrical stimulation studies, with timing around ankle push-off to exploit the addition of biomechanical torque. We also investigated the biomechanical effects of electrical stimulation on muscle tension throughout the gait cycle using Achilles tendon tensiometry [24]. We hypothesized that electrical stimuli would elicit increases in plantarflexor load at all gait phases, but with varying sensitivity, and with the greatest mechanical effect around the timing of entrainment. Finally, we investigated whether treadmill walking or overground walking entrains more persistently to rhythmic electrical stimulation. We hypothesized that overground walking would demonstrate more persistent entrainment, as observed with Anklebot [14].

The overall aim of this study was to determine to what phase of the gait cycle rhythmic electrical stimulation of the medial gastrocnemius entrains, as well as the effects of treadmill vs. overground conditions and stimulus amplitude. We observed gait entrainment in both treadmill and overground conditions, with more prevalent entrainment in treadmill walking. Phasing of the entrained stimuli was bimodal, aligning with either ankle push-off or foot contact for both treadmill and overground walking. In addition, we confirmed the existence of a mechanical effect, with Achilles tendon wave speed (loading) significantly different depending on the gait phase timing of electrical stimulation, however a mechanical effect at ankle push-off was not found.

## Methods

### Ethics statement

Eight non-impaired college-aged women were recruited to participate in the treadmill entrainment portion of this study. Six also completed the overground entrainment portion and five of the women also participated in the muscle force portion of the study. All participants reported no neurological or biomechanical impairment at enrollment. The study was approved by the University of Wisconsin Madison – Madison Health Services Institutional Review Board (IRB), approval number 2017-0705. Subjects gave their written informed consent to participate using IRB-approved procedures.

### Experimental protocols

#### Entrainment protocol

**Fig 1:**
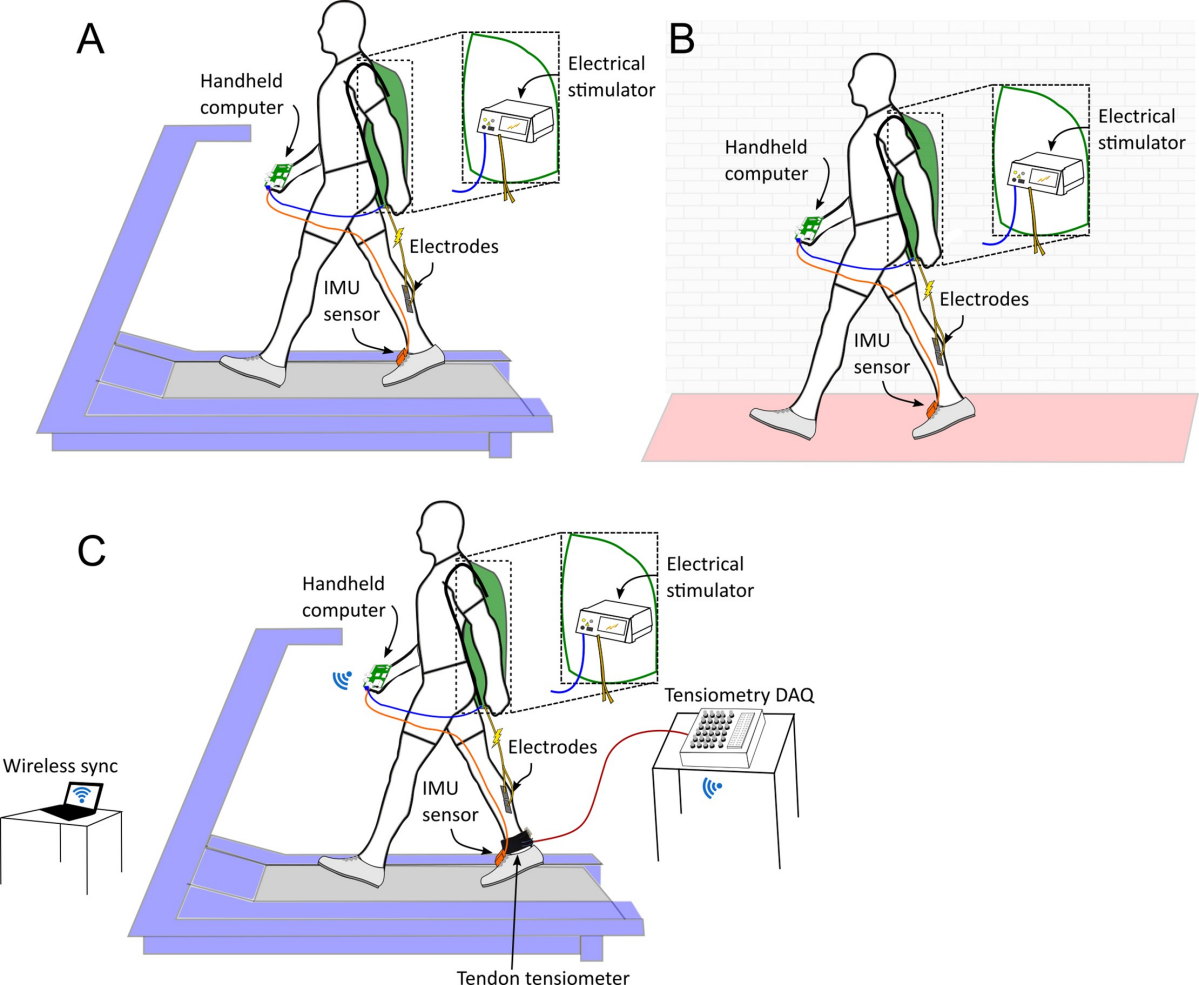
Setup diagram for experimental protocols. A and B show the treadmill and overground walking entrainment experimental setups. The electrical stimulation (ES) device sits inside a drawstring backpack and connects to a handheld computer. An IMU sensor on the shoe also connects to the handheld computer to determine stride period from foot movement. The computer commands rhythmic ES to the right medial gastrocnemius muscle at the subject’s observed stride period. C shows the additional equipment used to test electromuscular response during electrical stimulation. In addition to the ES device, IMU sensor, and handheld computer, a tendon tensiometer is worn on the Achilles tendon and records data through a desktop data acquisition system. All equipment is synchronized wirelessly and ES is applied to the right gastrocnemius muscle at unpredictable gait phases while the subject walks on a treadmill.

Subjects wore inertial measurement units (IMU; 3-Space Data Logger High-G; Yost Labs, USA) on the insteps of both shoes to calculate gait parameters. The IMUs logged data to onboard memory at a sampling frequency of 200 Hz. The right shoe IMU was connected via USB to a handheld computer (Raspberry Pi 3B) to stream real-time raw data. The IMU and handheld computer system times were synchronized before data collection. The electrical stimulation unit (RehaStim, Hasomed GmbH, Germany) was also connected to the handheld computer via USB, and was set to deliver electrical stimulation pulse trains (350 μs pulses at 40 Hz for 0.1 s) in a biphasic pattern with current amplitude control. Two surface electrodes were positioned so the right medial gastrocnemius muscle was between them according to the stimulator’s user instructions; we chose the medial gastrocnemius because of its strong plantarflexor function and ease of stimulation. Stimulation times from the handheld computer and stimulation signals recorded on a different computer system confirmed that the signals did not drift during the recording periods. To determine the stimulation amplitude, the subjects sat with their legs relaxed and the stimulation began at 10 mA and increased in steps of 2 mA until an observable heel lift occurred (minimum motor threshold). Next the stimulation amplitude continued to increase in steps of 2 mA until the subjects determined their maximum tolerable amplitude (just below pain threshold). The amplitude used for the walking entrainment trials was 2/3 between the thresholds (*Entrainment* stimulus). Across subjects, the *Entrainment* amplitude ranged from 16-31 mA, with a mean of 25.25 ± 5.62 mA (see Table 1). The overall setup is shown in the top of Fig 1.

**Table 1:** Subject stimulation amplitudes, participation, and entrainment results.

	*Entrainment* Amp	Treadmill Entrainment?	Overground Entrainment?	Muscular *Min* Amp	Muscular *Mid* Amp	Muscular *Max* Amp
**S1**	30 mA	Yes	N/A	18 mA	45 mA	60 mA
**S2**	26 mA	Yes	N/A	22 mA	27 mA	30 mA
**S3**	18 mA	No	No	N/A	N/A	N/A
**S4**	16 mA	Yes	No	20 mA	25 mA	28 mA
**S5**	30 mA	Yes	Yes	16 mA	23 mA	28 mA
**S6**	24 mA	Yes	Yes	20 mA	24 mA	27 mA
**S7**	31 mA	Yes	Yes	N/A	N/A	N/A
**S8**	27 mA	Yes	Yes	N/A	N/A	N/A

**Fig 2:**
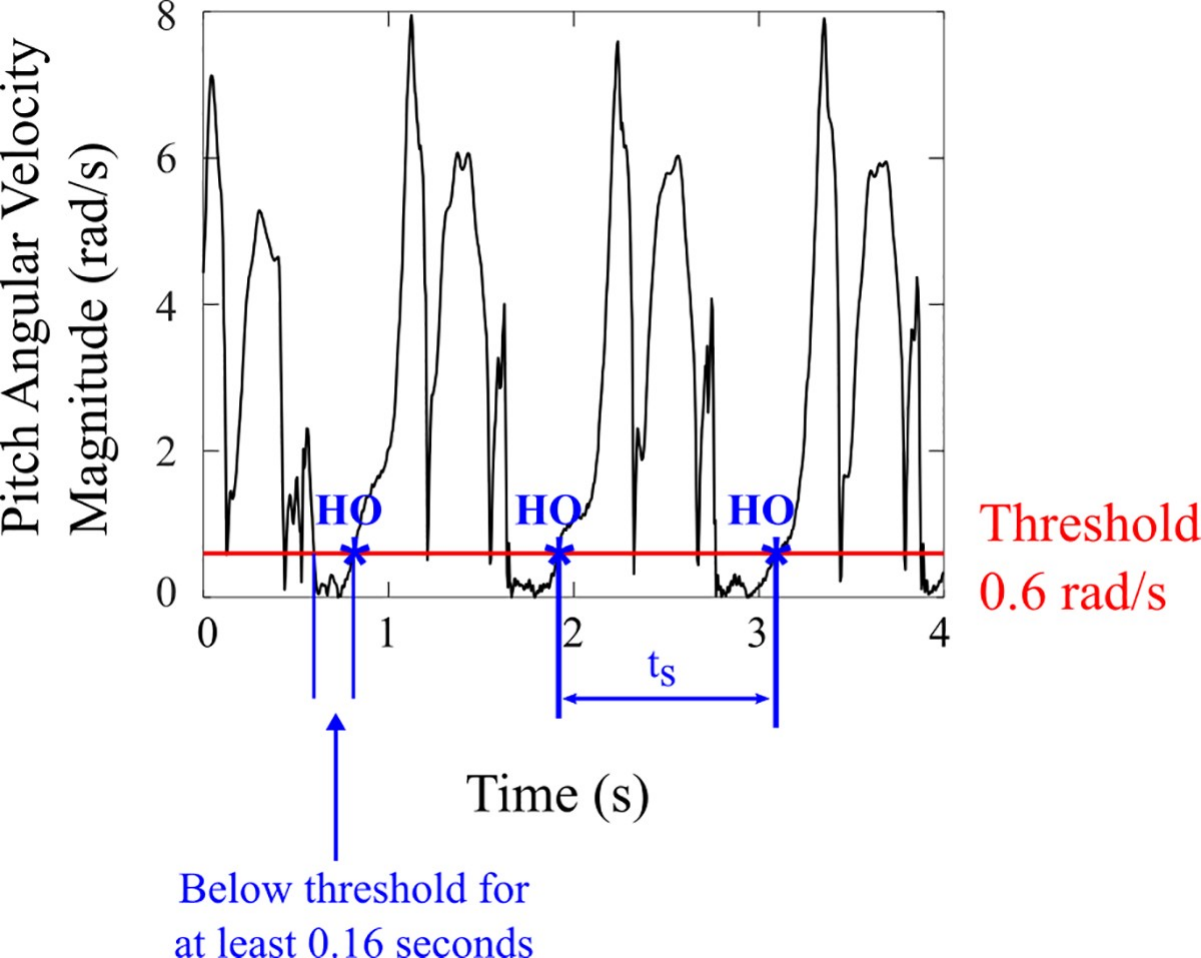
Real-time stride period calculation. The magnitude of pitch angular velocity is used to detect strides. Heel-off (HO) is detected when pitch angular velocity magnitude exceeds the threshold after it was below the threshold for at least 0.16 s. Stride period (t_s_) is the time between consecutive HO’s and is averaged over the first 10 s of each trial.

The protocol for treadmill walking (all eight subjects) included four trials (although the first subject completed six trials) of two minutes each walking at the subject’s self-selected constant speed (Experiment A). We chose four trials of two minutes each so we could observe entrainment from multiple initial phases of stimulation from each subject, while limiting the total testing time to avoid fatigue. Subjects started the treadmill and set it to a speed of their choice. Once settled at this steady speed, the subjects pushed a button on the handheld computer to start the trial. The average stride period was calculated from successive heel-off events over the next 10 seconds using a threshold on pitch angular velocity magnitude of the foot-mounted IMU to segment strides (Fig 2). The IMU’s then began logging data, and a random delay of 0-1 seconds was employed before the start of electrical stimulation so that it began at different phases in the gait cycle on each trial. The stimulation period was set to the calculated average stride period, and the amplitude was set at the *Entrainment* level determined above. At the end of two minutes, the subject was instructed to push the button and stop the treadmill to end the trial. The other three trials were repeated at the same treadmill speed, with short breaks between trials (1 minute).

The protocol for overground walking (six subjects) included four trials of two laps each in a rectangular hallway path (Experiment B). Subjects began walking at a constant, self-selected speed, then pushed the button to start the trial and again to end it after two laps. As above, stride period was averaged over the first 10 seconds and the electrical stimulation was applied at the calculated stride period and *Entrainment* level amplitude. For both parts of the study, the electrical stimulation times were saved to a file on the handheld computer, using the same time reference as the IMU data.

#### Muscle force protocol

To determine the biomechanical effects of electrical stimulation (ES), five subjects returned on a separate day for a second experiment in which muscle force was estimated during normal strides and strides with electrical stimulation during different phases of the gait cycle (Experiment C). Subjects performed one walking trial with each of three stimulation magnitudes, with intermittent, randomized stimulation timing (details below). In addition to the instrumentation used in the entrainment trials, we used tendon tensiometry [24] to estimate changes in Achilles tendon wave speed as an indicator of tendon loading and plantarflexor muscle force. The tensiometer uses micron-scale taps and skin-mounted accelerometers to track shear wave propagation speed along the tendon, with higher wave speeds indicating higher tendon tension [24]. Subjects wore a shear wave tensiometer over the right Achilles tendon, connected to a benchtop driver and data acquisition system. All measurement systems were synchronized wirelessly with the motion capture system. The overall setup is shown in Fig 1C.

Each subject repeated the stimulation amplitude calibration described above to determine new values for minimal motor threshold (*Min*, range 16-22 mA, mean 19.2 ± 2.28 mA*)*, maximum tolerable amplitude (*Max*, range 27-60 mA, mean 34.6 ± 14.24 mA*)*, and 2/3 between *Min* and *Max* (*Mid*, range 23-45 mA, mean 28 ± 9.12 mA*)* electrical stimuli. Amplitude values across subjects are shown in Table 1. These three levels of stimulus were presented to the subject, one trial each at a constant amplitude, with the amplitudes in random order. In each amplitude condition, the subject walked on a treadmill at their self-selected walking speed as in Experiment A. The subject pushed a button once they reached their chosen walking speed, and the average stride period was calculated over 10 seconds using a threshold on the right foot IMU pitch angular velocity magnitude to segment strides.

**Fig 3:**
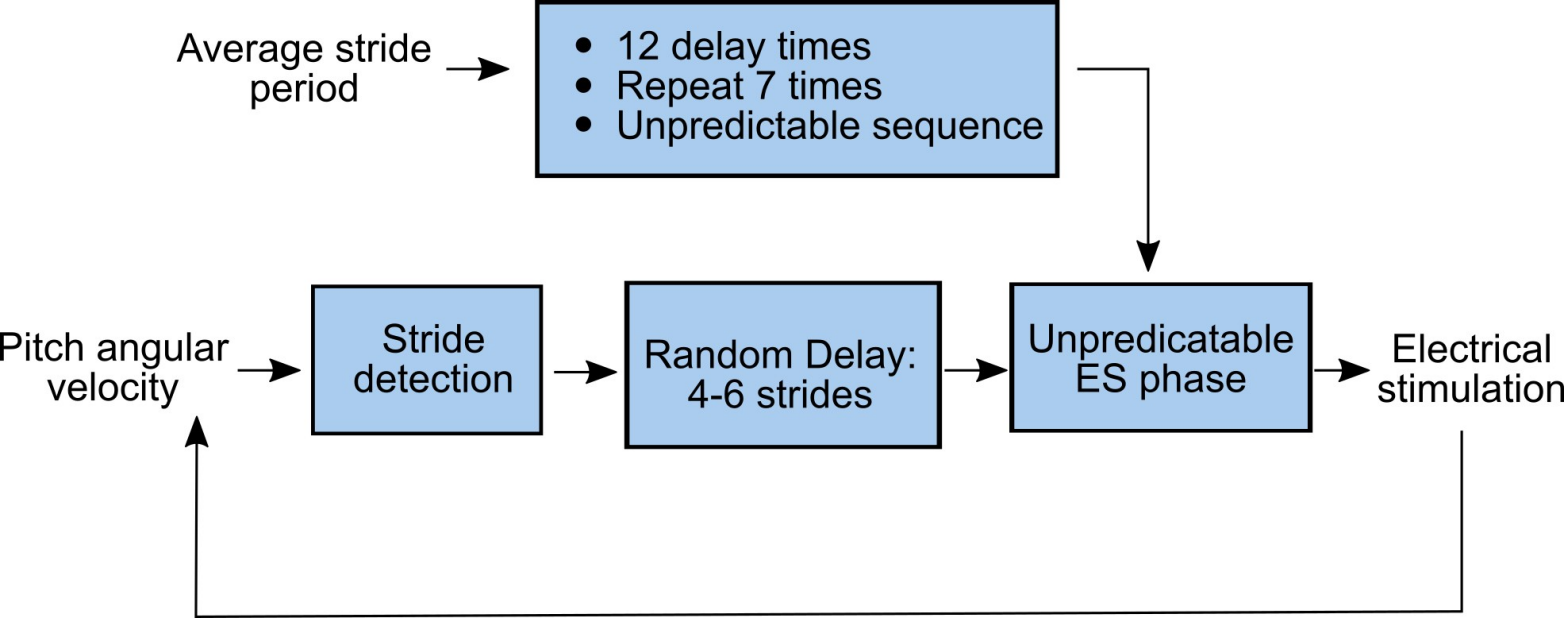
Flow chart for applying ES at unpredictable gait phase (Experiment C). Average stride period over the first 10 s is used to create delay times to test approximately 8 gait phases during stance and 4 gait phases during swing, each repeated 7 times and in random order. Real-time pitch angular velocity is used to detect strides. After a random delay of 4-6 strides the electrical stimulation (ES) is applied to the right gastrocnemius muscle at the next gait phase in the sequence. The same procedure to calculate delays and stimulus phases was used for all three stimulus amplitudes.

The average stride period was used to create an array of delay times to ensure unpredictable timing of the stimuli. Delays were implemented as a wait time (0 to 1 stride periods) after stride detection by the IMU. Delays were chosen to achieve stimulation during 8 distinct sub-phases within stance phase (0-49% of the gait cycle in 7% increments, and 49-60%) and 4 sub-phases within swing phase (60-100% in 10% increments). This array was repeated 7 times (for a total of 84 electrical stimulation pulse trains per trial), and the array was randomized and saved. Each stimulation pulse train was separated by a random integer (from 4 to 6) of normal strides. After each stimulus, the controller waited for the designated number of strides and then the designated delay time, and then applied the electrical stimulation pulse train. A trial ended after completing the 84 electrical stimulation pulse trains; total trial duration was approximately 10 minutes (dependent on self-selected constant speed). Then, the next trial used a different (randomized) amplitude, with the same procedure to calculate the sequence of delays and gait phases. The general protocol is shown in Fig 3.

### Data analysis

#### Entrainment data

For the entrainment tests (Experiments A and B), we combined data from the IMU and electrical stimulator to evaluate the presence, absence and persistence of gait entrainment in each treadmill and overground entrainment trial. The IMU data were processed in MATLAB (The Mathworks, USA) using thresholds on angular velocity and acceleration magnitudes to segment strides using algorithms previously developed [25]. This method gave approximate stationary period times to segment strides. To find heel-strike (HS) and toe-off (TO) times, the pitch angular velocity data were used to find the prominent peaks during each stride. The first major positive peak of each stride represents TO and the second major positive peak represents HS. Each HS to subsequent HS represents 0-100% of a gait cycle. Right foot HS (RHS) to left foot HS (LHS) and LHS to RHS times for five subjects during treadmill walking were used to determine whether the unilateral stimulation caused a limp. Using a two-sample one-tailed *t*-test (significance level *α* = 0.05; mean 458 strides per subject), only two of the five subjects had asymmetric step times (LHS to RHS significantly greater than RHS to LHS), and the differences in step times for those subjects were only 1.95% and 2.18%. This difference was deemed negligible, so only the RHS was used in calculating the results, and is referred to as HS in the following sections.

**Fig 4:**
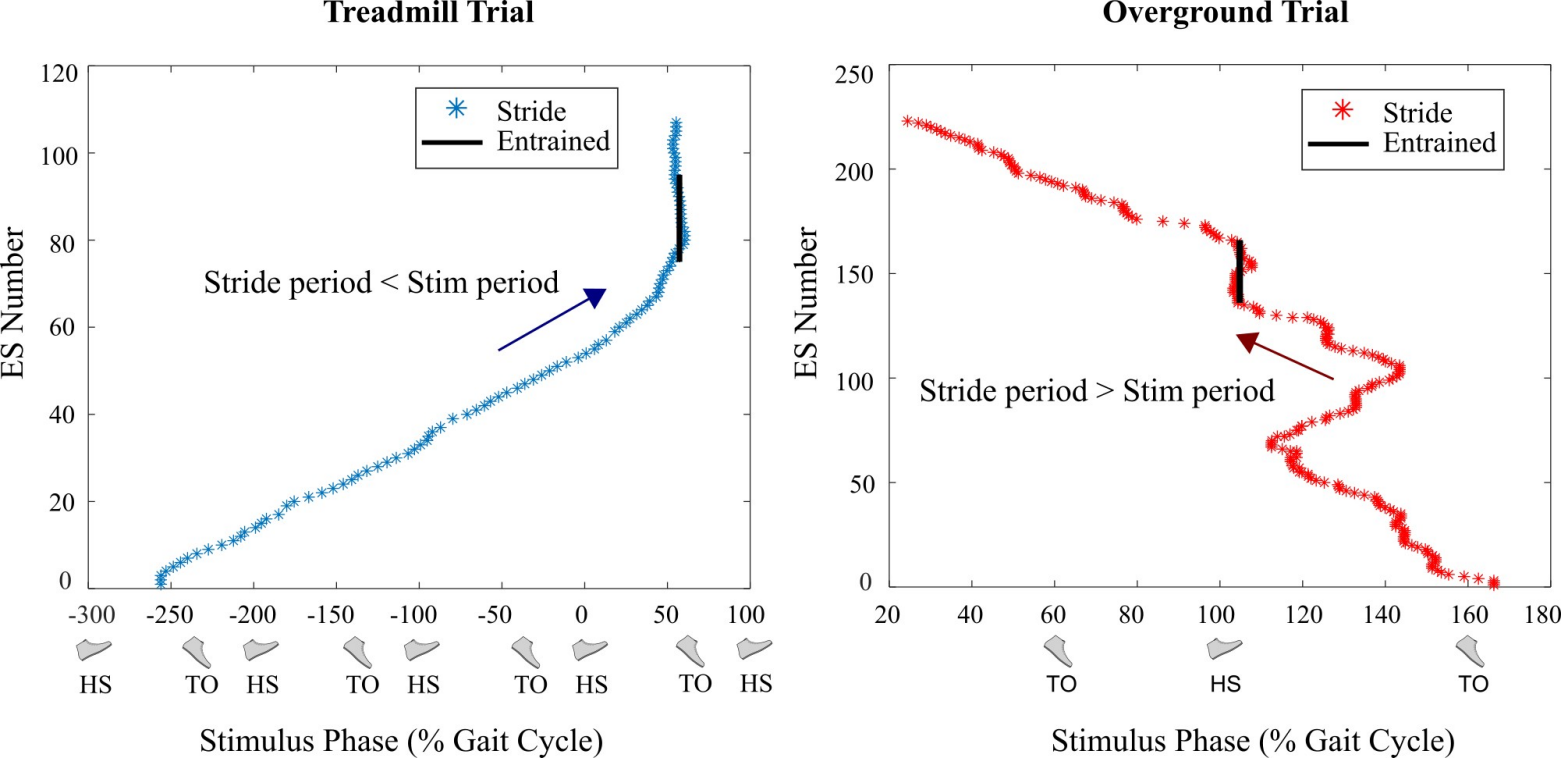
**Examples of trials with entrainment of gait (Experiments A and B).** The sequence of stimuli advances along the vertical axis, with the gait phase of each stimulus plotted on the horizontal axis. The final stimulus is placed within the 0-100% range of gait phase, and phase is unwrapped across gait cycles to show continuous drift. The left graph shows a two-minute treadmill trial that entrained to the ES (Experiment A). Successive stimuli drift later in the stride (a positive slope), indicating that the stride period of the subject before entrainment was less than the stimulation period. The right graph shows a two-lap overground trial that entrained to the ES (Experiment B). Successive stimuli drift earlier in the stride (a negative slope), indicating that the stride period before entrainment was greater than the stimulation period. The regions where entrainment occurred (gait phase within +/- 3% for 20 consecutive strides) are represented by a black vertical line.

The start time of each electrical stimulus was compared to the preceding and following HS times to determine its timing as a percentage of gait cycle. For each trial we plotted stimulus number vs. the gait phase of each stimulus to observe entrainment and phase drift (see examples in Fig 4). A positive slope occurs when the stride period is less than the electrical stimulation period, causing the stimulus to drift later in sequential strides; a negative slope occurs when the stride period is greater than the electrical stimulation period, causing the stimulus to drift earlier in sequential strides. During periods of entrainment, the gait phase of the stimulus is stable with only minor variations. We defined periods of entrainment as 20 or more successive strides with gait phase of the electrical stimulation within ± 3% of the gait cycle; this threshold was chosen as a restrictive definition of entrainment approaching the time resolution of the equipment, to avoid false positives for entrainment detection.

For entrained segments of all trials, we determined the dominant gait phases of stimuli within the gait cycle. We plotted stimulus phases as a frequency histogram and fitted a kernel smoothing function to the histogram in MATLAB. We identified the peaks of the fitted curves and recorded the locations (in percent gait cycle) of the peaks as the dominant gait phases of stimulus entrainment.

A comparison of entrainment results between treadmill walking and overground walking was performed using the entrainment criteria described previously. We computed a two proportion z-test (significance level *α* = 0.05) for subjects entrained, trials entrained, and strides entrained. In addition, because entrainment occurs only when stimulus stride period is very close to stride period [13, 21], we evaluated whether the stimuli themselves affected stride period for treadmill and overground walking. To determine how well subjects matched their stride period to the stimulation period, we computed the stride period mean squared error (SPMSE) relative to the stimulation period according to Eq 1,

$$SPMSE=\frac{1}{n-1}\sum_{i=1}^{n}{\left(t_{s,i}-t_{\mathrm{stim}}\right)}^{2}$$ (1)

where *n* is the number of strides with stimulation in the trial, *t*_s,i_ is the stride period of the i^th^ stride, and *t*_stim_ is the electrical stimulation period.

#### Muscle force data

For the electromuscular response tests (Experiment C), we combined IMU, stimulator and tensiometer data to evaluate the effects of electrical stimulation of the medial gastrocnemius on Achilles tendon loading. The IMU data were processed as described for the entrainment experiment. The times of the electrical stimuli from the handheld computer file were compared to the wave speed times collected from the tendon tensiometer. The effect of electrical stimulation on wave speed could then be observed.

**Fig 5:**
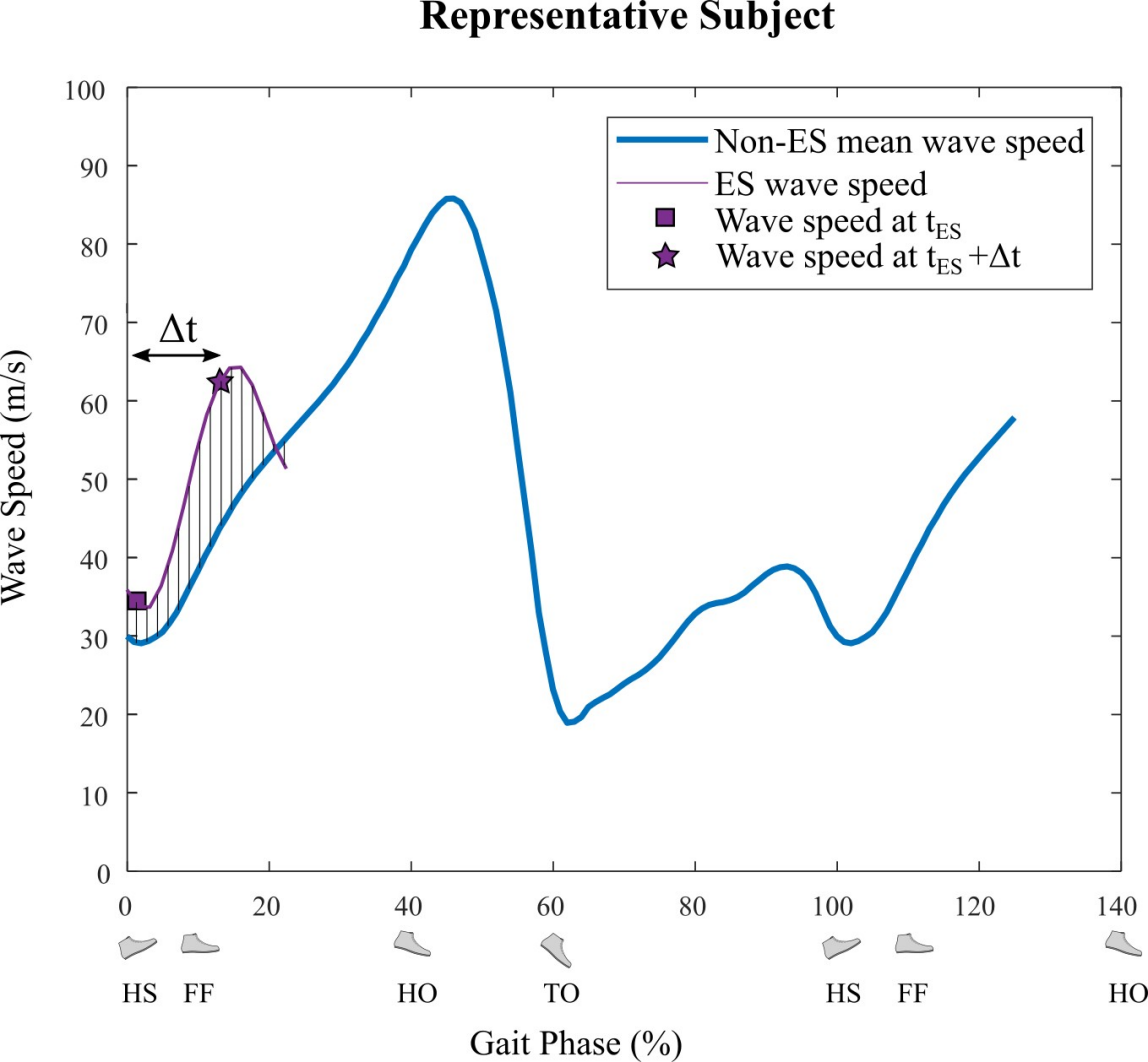
Calculating Δ*t* for maximum effect from ES (Experiment C). The blue curve shows the mean Achilles tendon wave speed for all non-ES strides for a representative subject. The purple curve shows a portion of wave speed for an ES stride (ends 0.3 seconds after ES). The square denotes the time and wave speed when ES was applied. The black lines show the difference between ES and non-ES wave speed, and the star shows the time and wave speed when the difference between the ES wave speed and the non-ES wave speed was greatest. Overall $\bar{\Delta t}$ was calculated as the mean of individual Δ*t* values from all ES across all subjects ($\bar{\Delta t}$ = 0.1465 s).

To account for the electromuscular delay between the time of the stimulus and its resulting force development, wave speed at times from 0-0.3 s after each stimulus were calculated. The upper limit of 0.3 s was chosen to be sufficiently greater than electromuscular delay times reported in literature [26, 27]. This perturbed wave speed was then compared to the average wave speed value at the same gait phase for strides without electrical stimulation (Fig 5) during that time interval (0-0.3 s from ES). For each stimulus we measured the time that maximized the difference between electrical stimulation wave speed and non-electrical stimulation wave speed, Δ*t*. The mean time of maximum difference was $\bar{\Delta t}$ = 0.1465 s (SD ± 0.0163 s across all ES strides from all subjects). All subsequent measurements comparing electrical stimulation wave speed to non-electrical stimulation wave speed were made at time $t_{\mathrm{ES}}+\bar{\Delta t}$, where *t*_ES_ is stimulus time.

To compare Achilles tendon wave speeds with vs. without electrical stimulation, strides with similar stimulation timings were grouped together. The 12 timing bins represented the 8 targeted timings during stance and 4 timings during swing phase. To compare wave speed values from different subjects, wave speed was normalized to the mean peak (push-off) wave speed of all non-ES strides in a specific walking trial. The recorded normalized wave speed following each stimulus at time $t_{\mathrm{ES}}+\bar{\Delta t}$ was paired with the mean wave speed at the matched gait phase across all non-ES strides of that subject. We computed a two-sample two-tailed *t*-test (significance level *α* = 0.05) of these wave speeds (with ES vs. mean without ES) in each bin of the gait cycle, at each amplitude of stimulation.

#### Stride period

We also investigated the effect of electrical stimulation timing on stride period. Since electrical stimulation applied just before foot contact will also affect the next stride, the stride period of each ES stride and the subsequent stride were averaged to find the stride period attributable to each stimulus. To compare values from different subjects, stride period was normalized to the mean stride period for all non-ES strides (also excluding strides immediately following ES). A statistical analysis was performed by comparing the normalized stride periods for all ES strides within each ES timing bin to the normalized mean and standard deviation of stride period in non-ES strides. We computed a two-sample two-tailed *t*-test (significance level *α* = 0.05) of normalized stride periods with ES compared to stride periods from non-ES strides, for each bin of ES timing.

## Results

### Entrainment

**Fig 6:**
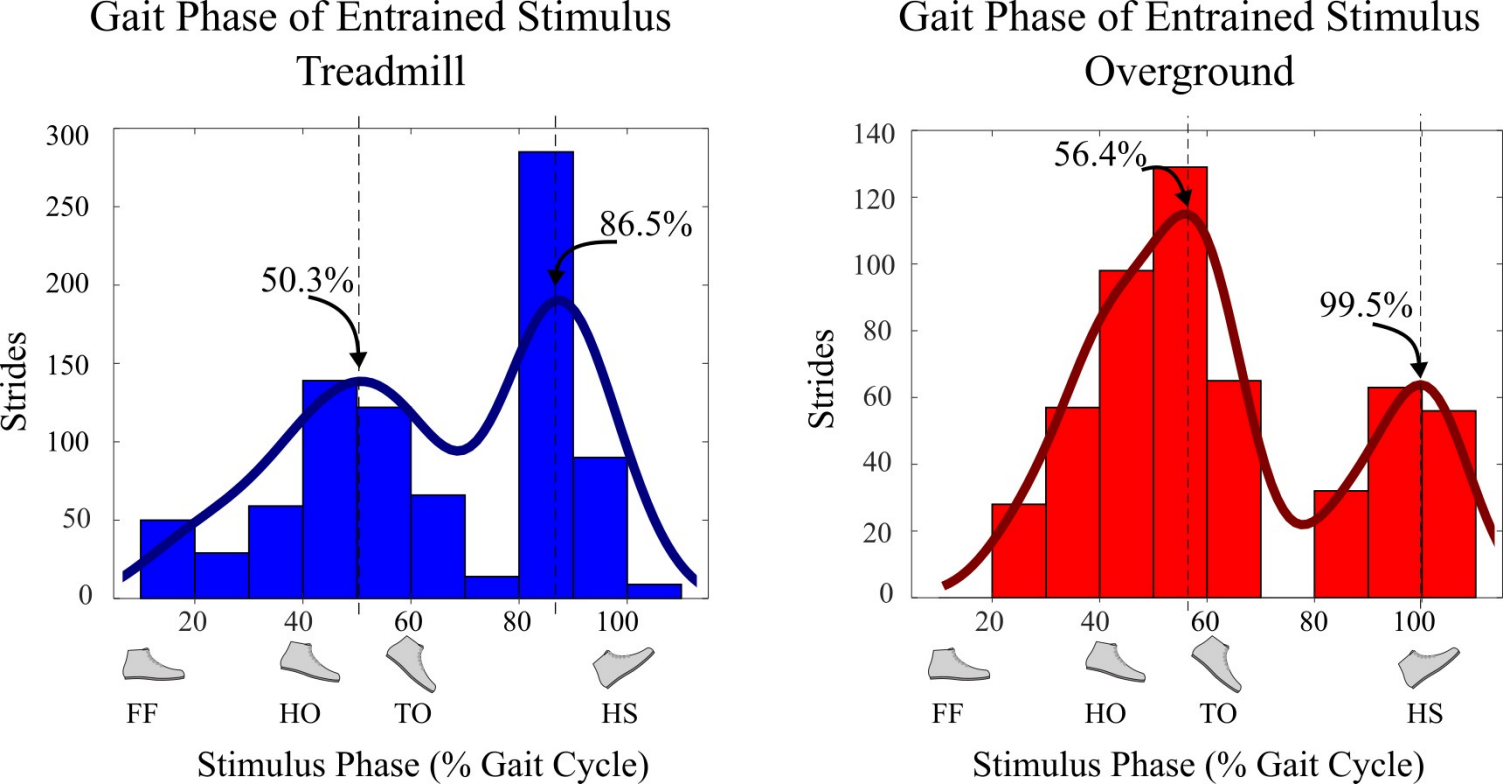
**Histograms of gait phase of electrical stimulus during entrained portions of treadmill (Experiment A) and overground walking (Experiment B).** The left graph shows the distribution of gait phase of ES for entrained strides during treadmill walking and the right graph shows the distribution of gait phase of ES for entrained strides during overground walking. The curves show the kernel smoothing function fit for the histograms, and the locations of the peaks based on the fitted curves are denoted with vertical lines (in percent gait phase). For both treadmill and overground walking conditions, the gait phase of ES for entrained strides primarily occurred just before toe-off (TO) and heel strike (HS).

Seven of eight treadmill walking subjects and four of six overground walking subjects showed gait phase entrainment to rhythmic electrical stimulation (Table 1). Histograms of the distribution of gait phase of electrical stimulation in entrained strides revealed a bimodal distribution of phase alignment. For both treadmill and overground walking conditions, the dominant phase alignments occurred roughly around ankle push-off and just before heel strike (Fig 6). For treadmill walking, the dominant phase alignments were 50.3% and 86.5%, and for overground walking the dominant phase alignments were 56.4% and 99.5%. We observed that most positive slope trials (stride period shorter than stimulation period) entrained around push-off and negative slope trials (stride period longer than stimulation period) entrained around foot contact. Using the net direction of drift (as in Fig 4) in the 20 strides immediately before each bout of entrainment as an indicator of this phenomenon, 15 of 18 bouts of entrainment with mean gait phase near toe-off (40-70%) had shorter stride periods compared to stimulation period, and 6 of 11 bouts of entrainment with mean gait phase approaching or just after heel-strike (80-110%) had longer stride periods compared to stimulation period. This relationship was even more pronounced when only considering treadmill walking trials, with shorter stride periods for 9 of 10 bouts of entrainment with mean gait phase near toe-off and longer stride periods for 4 of 6 bouts of entrainment with mean gait phase near heel-strike.

**Fig 7:**
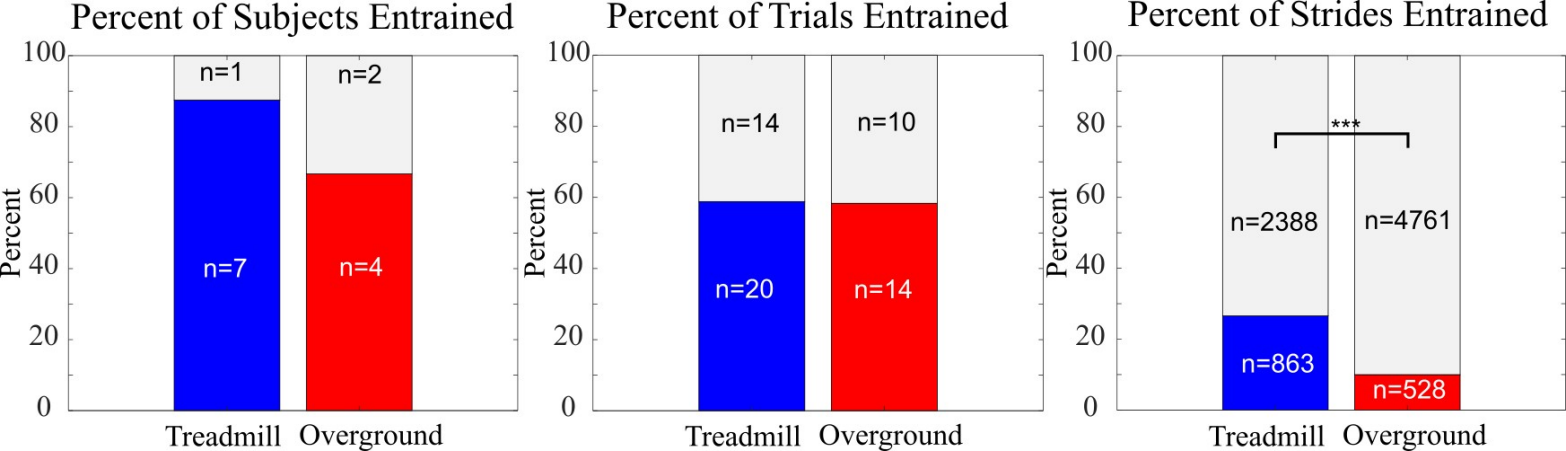
Percent of entrainment for treadmill (Experiment A) and overground (Experiment B) trials. The left graph shows that a greater percentage of subjects entrained to the electrical stimulus during treadmill walking than during overground walking. The middle graph shows that a similar percentage of trials exhibited entrainment in both conditions. The right graph shows that the percentage of total strides that occurred during periods of entrainment was significantly higher in treadmill than in overground walking (p < 0.001). Numbers in the bars indicate how many subjects, trials and strides were or were not entrained.

**Fig 8:**
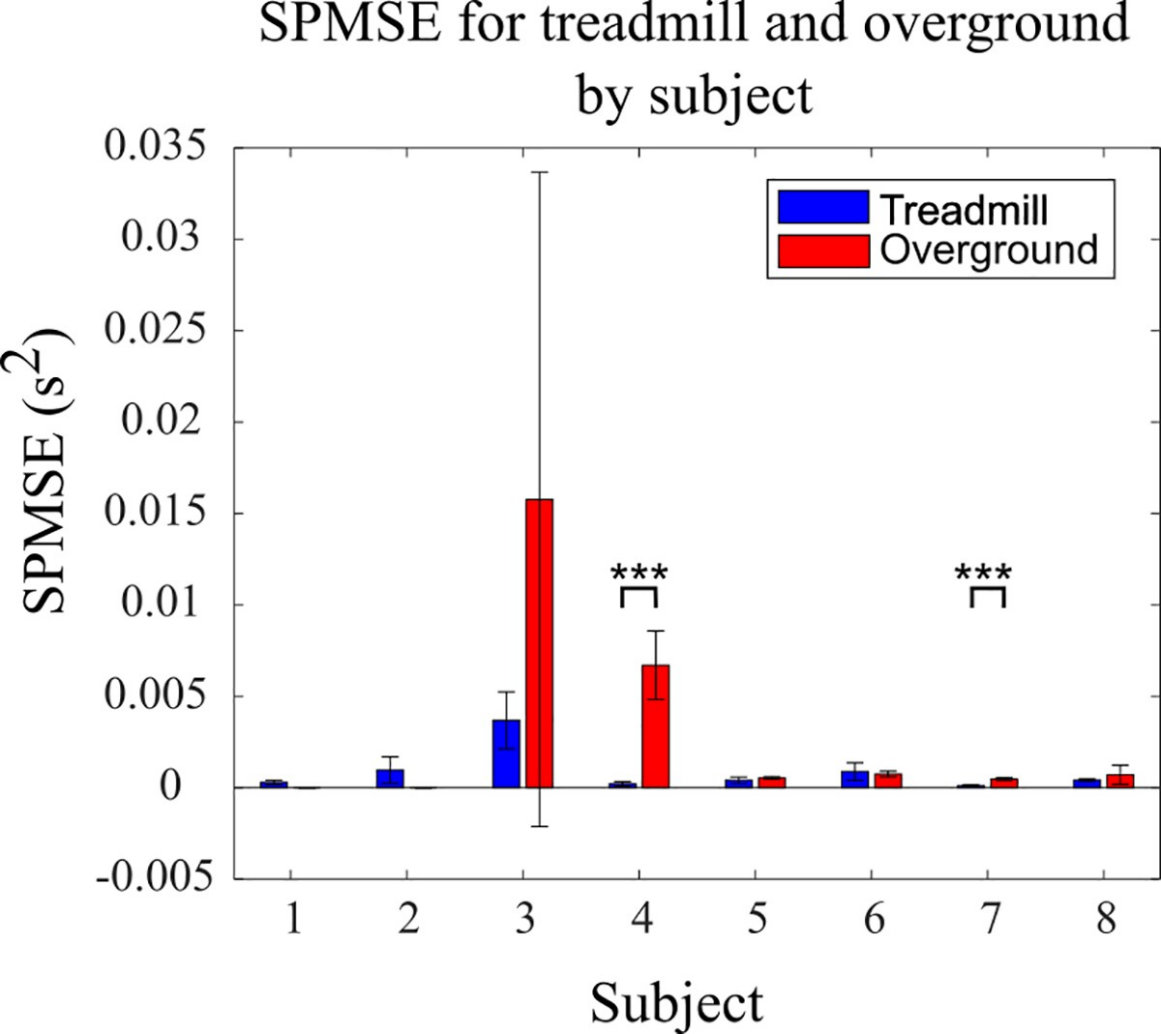
**Stride period variation from stimulation period (Experiments A and B).** The graph shows the stride period mean square error (SPMSE) from the preset stimulation period for each subject’s treadmill and hallway trials. The *** represents p < 0.001. In general, the SPMSE was greater during overground walking, although not significant when combining all subjects.

In general, entrainment occurred more frequently for treadmill walking than for overground walking, as shown in Fig 7. The result was only statistically significant when comparing the proportion of strides entrained for the treadmill and overground walking conditions, with no statistically significant differences when comparing the proportion of subjects entrained or the proportion of trials entrained. It was checked whether the discrepancy in the percentage of strides entrained in treadmill vs. overground walking could be explained by a systematically greater difference between stride period and stimulation period in the overground condition. Overall, the stride period mean-squared error (SPMSE) was greater for overground trials, although only significantly greater for two of the subjects (Fig 8). This trend persisted after removing the extreme values of Subjects 3 and 4, although again without statistical significance.

#### Muscle force

**Fig 9:**
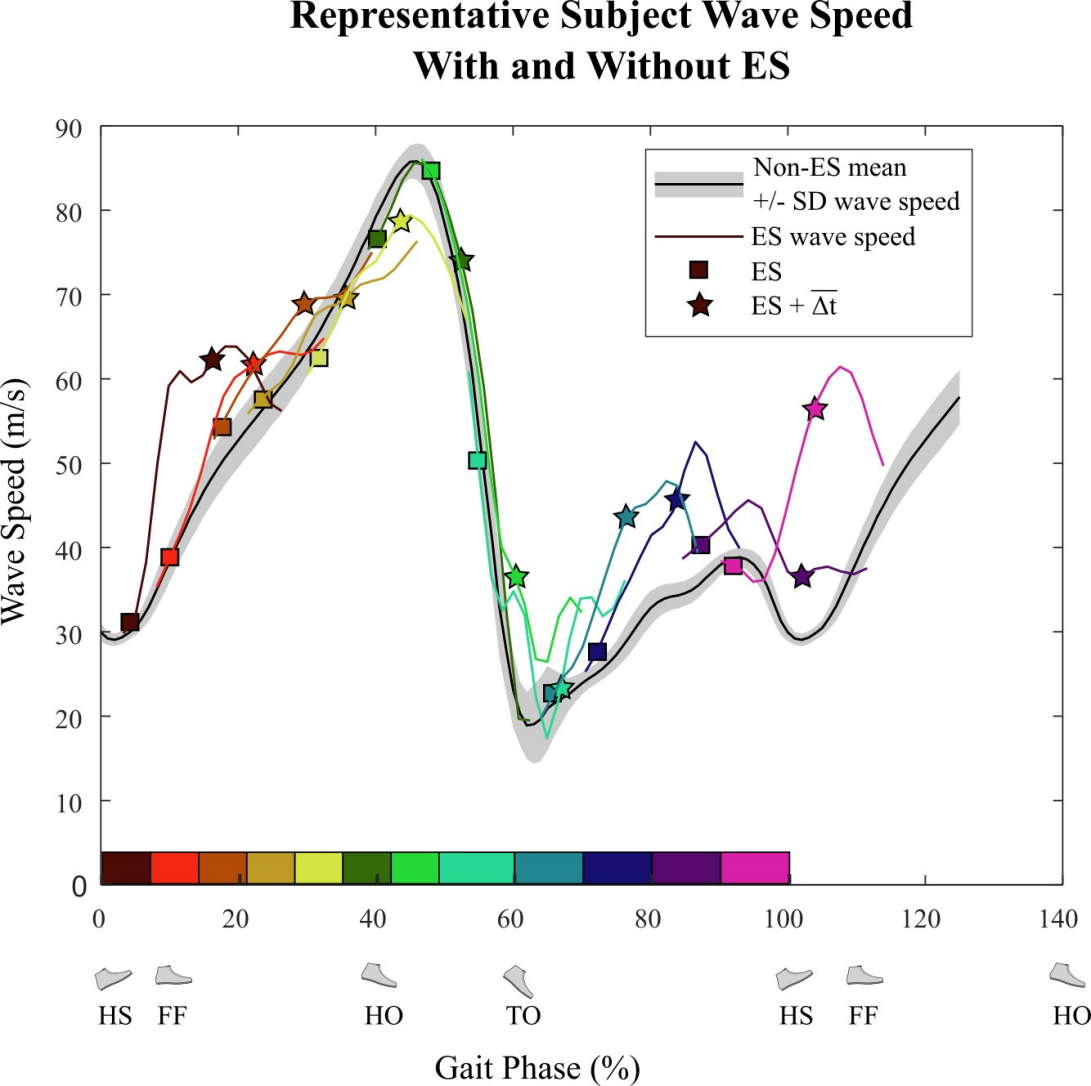
Achilles tendon wave speed from a representative subject with and without ES (Experiment C). The black curve is the mean wave speed for all non-ES strides and the grey shading is the standard deviation band. The colored curves show local deviations in wave speed after 12 ES were applied – 8 during stance and 4 during swing. The squares show the start of ES and the stars show the wave speed after $\bar{\Delta t}$ (0.1465 s) for the ES stride of the corresponding color. Traces from each stimulus are shown from 0 to 0.3 s after the time of each stimulus.

**Fig 10:**
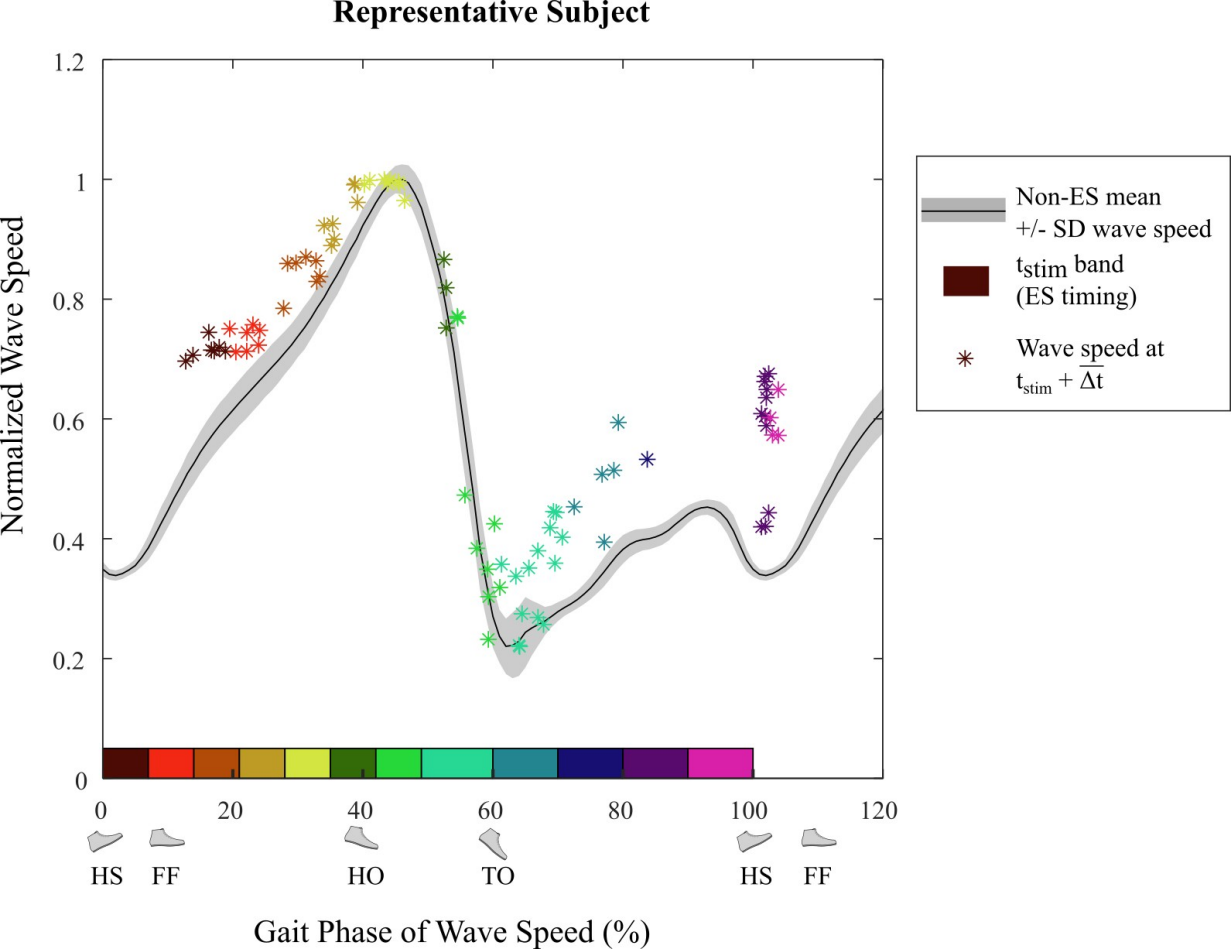
Representative subject normalized Achilles tendon wave speed for grouped ES timing (Experiment C). The black curve is the mean normalized wave speed for all non-ES strides and the grey shading is the standard deviation band. Wave speed values are normalized relative to the subject’s mean peak wave speed for strides without ES. The colored boxes show the range of gait phase for each ES timing band, and the asterisk (*) of the same color represents normalized wave speed at $\bar{\Delta t}$ (0.1465 s) after each ES.

Four of the five subjects who completed Experiment C showed similar Achilles tendon wave speed patterns – an indicator of muscle-tendon loading – during gait. Overall, electrical stimulation in early stance and swing phase caused an increase in tendon wave speed after the electromuscular delay $\bar{\Delta t}$ (= 0.1465 s) compared to the tendon wave speed without electrical stimulation (Fig 9). The subject (S4 in Table 1) with a different pattern reported after testing that she was recovering from a recent bout of tendinitis. This subject was excluded from further data analysis. Fig 10 shows the relationship of normalized tendon wave speed values at time $\bar{\Delta t}$ after electrical stimulation (ES) compared to continuous wave speed measurements from strides with no stimulation (non-ES) for a representative subject.

**Fig 11:**
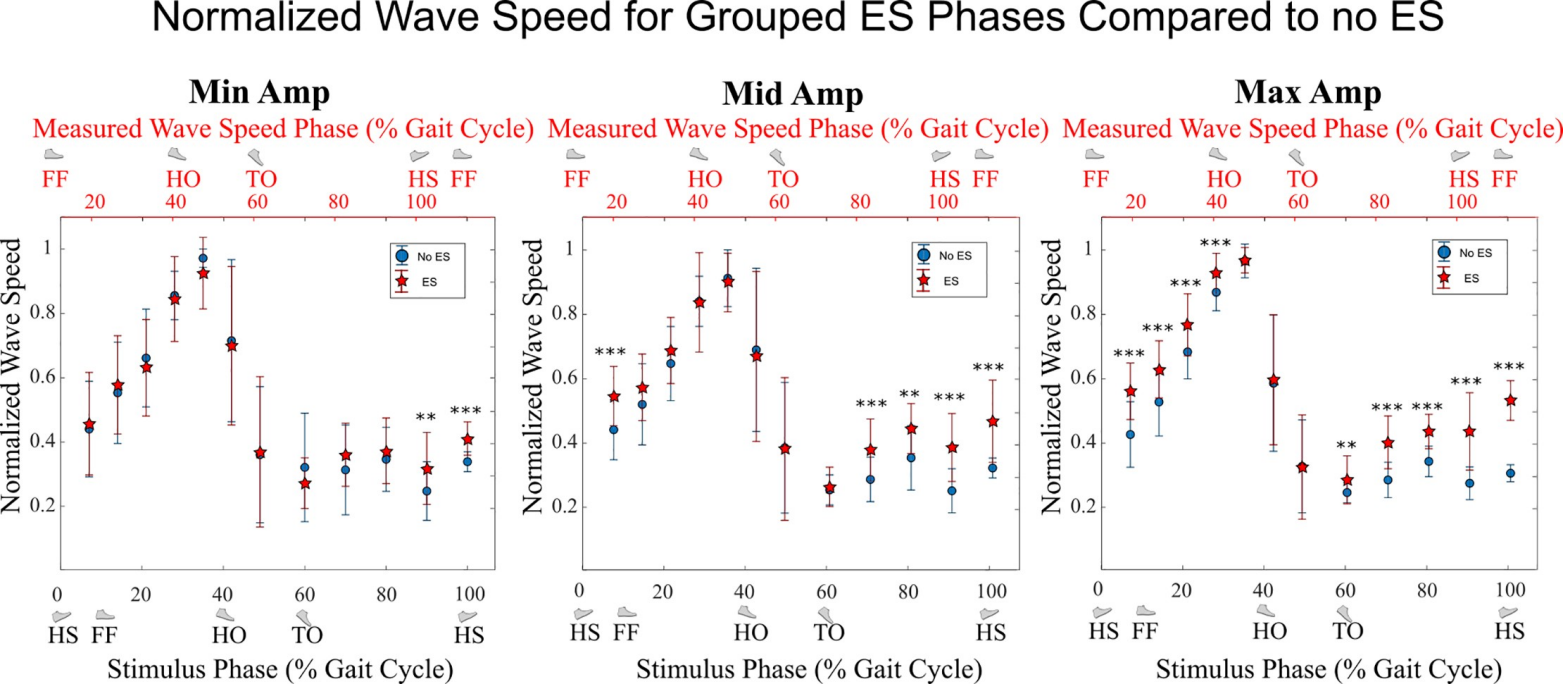
Normalized Achilles tendon wave speed with and without ES at different gait phases (Experiment C). Wave speed was higher with ES than without ES for most phases with Max stimulation (right graph), with effects diminishing at lower levels of stimulation. Wave speed values are normalized relative to each trial’s mean peak wave speed for strides without ES. The red stars show the mean normalized wave speed for all ES strides within each bin of gait phase, measured $\bar{\Delta t}$ after stimulation began. The corresponding blue circles show the mean normalized wave speed for all non-ES strides at the same time. Error bars show standard deviation. The bottom axis shows the gait phase of the ES; the x-value of each data point represents the end of the associated bin, and the upper axis shows the gait phase when the wave speed measurement was recorded. Symbols: * p < 0.05, ** p < 0.01, *** p < 0.001.

The number of ES that occurred in each timing bin (8 in stance, 4 in swing) varied from 10 to 47 (mean 26), with wave speed from each compared to the mean wave speed sampled at the matched phase from all non-ES strides (mean 1255 strides). With *Min* stimulation amplitude, the wave speed following ES was only significantly different (greater) when ES was applied in the 80-90% and 90-100% stride bins. With *Mid* stimulation amplitude, the wave speed for ES strides was significantly different (greater) when ES was applied in the 0-7%, 60-70%, 70-80%, 80-90%, and 90-100% bins. With *Max* stimulation amplitude, the wave speed for ES strides was significantly different (greater) when ES was applied in the 0-7%, 7-14%, 14-21%, 21-28%, 49-60%, 60-70%, 70-80%, 80-90%, and 90-100% bins. These results are summarized with *P*-values for each bin of gait phase in Fig 11.

#### Stride period

**Fig 12:**
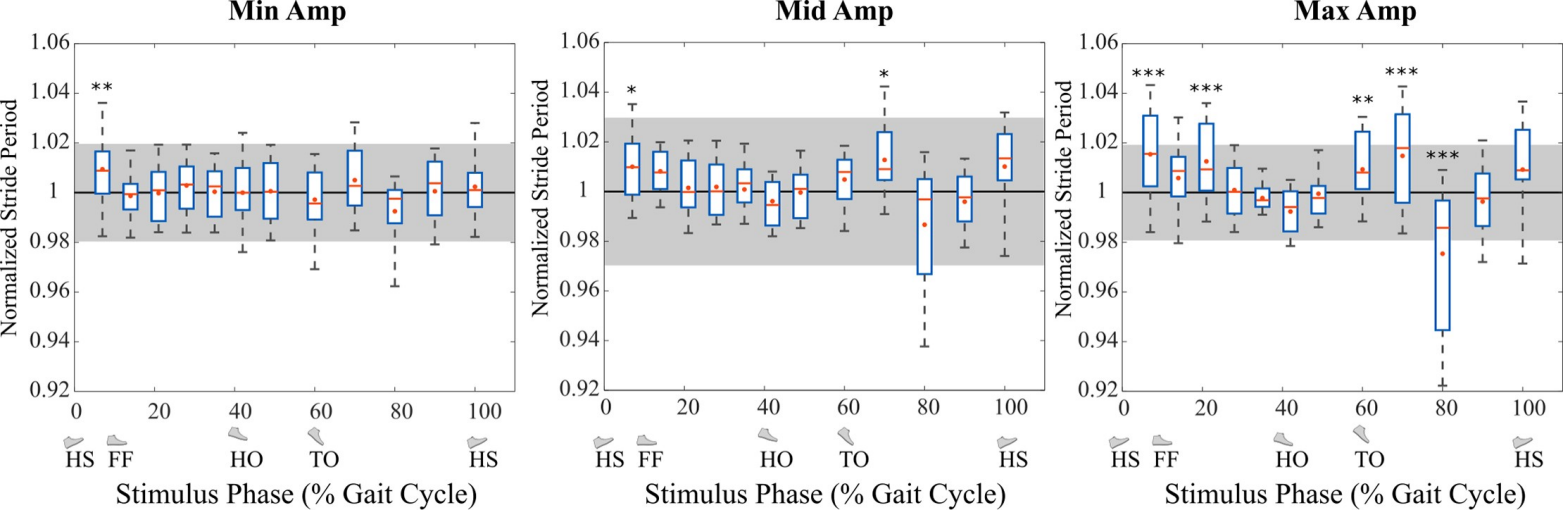
Normalized stride period for ES strides grouped by ES phase, compared to mean stride period for no-ES strides (Experiment C). ES led to altered stride period with the direction and magnitude of the change strongly dependent on the gait phase when ES was applied. The strongest effects were during early swing and early stance, especially with Max stimulation. Stride period is normalized relative to each trial’s mean stride period for strides without ES. The grey shading is the standard deviation band for strides without ES. The boxplots show normalized stride periods (mean of stride period for the ES stride and subsequent stride) for all ES strides within each bin ES initiation phase. The red line denotes the median and the red dot denotes the mean. The bottom and top edges of the boxes indicate the 25^th^ and 75^th^ percentiles. The bottom and top edges of the whiskers indicate the 9^th^ and 91^st^ percentiles. Symbols: * p < 0.05, ** p < 0.01, *** p < 0.001.

With *Min* stimulation amplitude, the stride period during electrical stimulation was only significantly different when ES was applied at gait phase 0-7% (greater). With *Mid* stimulation amplitude, the stride period was significantly different when ES was applied at gait phase 0-7% (greater) and 60-70% (greater). With *Max* stimulation amplitude, the stride period was significantly different when ES was applied at gait phase 0-7% (greater), 7-14% (greater), 14-21% (greater), 50-60% (greater), 60-70% (greater), and 70-80% (lesser). These results are summarized with *P*-values for each bin of gait phase in Fig 12.

## Discussion

The main purpose of this study was to determine whether the gait phase of rhythmic electrical stimuli applied to the medial gastrocnemius muscle converges to a gait phase just before toe-off where the stimuli assist propulsion, similar to results shown for rhythmic plantarflexion torque perturbation pulses [13, 14, 18]. The period of the electrical stimulation was set at approximately the natural stride period of subjects to determine the gait phase of stimuli that entrained strides converged to. For both treadmill and overground walking, the gait phase of the stimulus for entrained strides mostly occurred just before toe-off or just before heel strike (Fig 6). This observation does not appear to be due to different subjects having different preferred phase-locking. For the treadmill walking case, Subject 5 only entrained around toe-off and Subject 6 only entrained around heel strike, however all other subjects entrained to both cases. Similarly, for the overground walking case, Subject 5 only entrained around heel strike and Subject 8 only entrained around toe-off, and all other subjects entrained to both cases. This suggests the bimodal distribution is not due to differences between subjects. A hypothesis for the cause of this bimodal distribution, rather than convergence only at toe-off, is that the gait phase of the stimulus for entrained strides may depend on the subject’s stride period compared to stimulation period leading up to the entrainment. This phenomenon could be investigated explicitly using stimulation periods deliberately chosen to be different from the stride period, as in prior work [13, 14, 18]. We are planning such a test in a follow-up study.

The statistical analysis of stride period for ES applied throughout the gait cycle while walking on a treadmill (Fig 12) also seems to support this hypothesis. In the *Max* amplitude condition, when the ES was initiated at a gait phase of 49-60%, the stride period was significantly greater than the stride period without ES. Therefore, in entrainment trials where the stride period was less than the stimulation period, when the gait phase of the stimulus drifted toward toe-off the subject’s stride period may have increased and momentarily matched the stimulation period to cause entrainment of gait. The other entrainment case is also supported. With *Max* amplitude, when the ES was initiated at a gait phase of 70-80%, the stride period was significantly less than the stride period without ES. Stride period with ES remains lower (although without statistical significance) as gait phase approaches heel strike. Therefore, in entrainment trials where the stride period was greater than the stimulation period, when the gait phase of the stimulus drifted towards heel strike the subject’s stride period may have decreased and momentarily matched the stimulation period to cause entrainment of gait. Similar trends were observed in the *Mid* amplitude case but without statistical significance.

The sharp difference in stride period between stimuli in the 60-70% phase bin (longer stride period) vs. the 70-80% phase bin (shorter stride period) may indicate a phase threshold that drives entrainment to one or the other of the common timings. The reasons for such a threshold are not yet clear but they could be related to stable vs unstable responses to the input as observed in a modeling study of mechanical ankle perturbations [28]. This study used a simple state-determined model consisting of a point mass and rigid massless legs and feet to demonstrate entrainment of gait in response to superimposed periodic plantarflexion torque pulses. Perturbation to the trailing leg during stance accelerated the model, perturbation to the leading leg during stance decelerated the model, and perturbation during swing had no effect due to the massless legs [28]. The model imposed a constant stride length due to resetting the angle between the two legs for each step in the stride cycle, so depending on the phase of perturbation, the model speed and cadence could increase or decrease, leading to entrainment for perturbation periods both shorter and longer than the natural stride period. Stable entrainment regions occurred in negative slope regions of the average speed vs. phase of perturbation curve: the negative slope region with speed lower than natural speed just after foot contact was an attractor for entrainment to longer perturbation periods, and the negative slope region with speed greater than natural speed at the end of double stance was an attractor for shorter perturbation periods [28]. In this study, the *positive* slope regions for stride period vs phase of electrical stimulation in the 40-70% and 80-100% phase bins (Fig 12) may be similar attractors (model attractors had negative slope of speed vs. phase; stride period is the inverse of speed so we expect positive slope). The opposite result observed in this study, of shorter perturbation periods entraining near foot contact and longer perturbation periods entraining near push-off, may be related to the different walking conditions of a treadmill with constant speed and the model with constant stride length, as well as an effect during swing phase. For example, treadmill walking has been shown to reverse the sign of the correlation between speed and stride length due to the constant-speed constraint [29–31]. This effect could also underpin the different distributions of entrained gait phases in treadmill walking (mostly push-off) vs. overground walking (mostly heel strike). The opposite result from the modeling may also be caused by the stimulation periods for Experiments A and B being approximately equal to unperturbed stride period rather than systematically shorter or longer. We plan to address this question in a follow-up study.

Another key finding is that entrained strides occurred more frequently during treadmill walking than overground walking (Fig 7). This may also be explained by the fact that the stimulation period was set approximately at the subject’s stride period. Entrainment of gait requires the stride period to match the stimulation period (which is constant), but the statistical analysis of stride period for ES applied throughout the gait cycle shows that ES at certain times throughout the gait cycle will alter stride period (Fig 12). In overground conditions, this disturbance could accelerate or decelerate the subject depending on its phase within the gait cycle, causing the subsequent stimulation pulse to occur at an earlier or later gait phase and potentially altering walking speed. Walking on a treadmill forced the subjects to maintain a constant speed and therefore may have aided them in remaining at the stride period that matched the stimulation period. This idea is supported by past findings of very responsive coupling between speed and stride frequency in the face of perturbations [20, 32].

The other purpose of this study was to determine the electromuscular response to ES applied throughout the gait cycle. This was accomplished by measuring wave speed along the Achilles tendon, which is related to tension in the tendon [24] during Experiment C. The effect of ES on wave speed was greatest at $\bar{\Delta t}$ = 0.1465 +/- 0.0163 s after the start of ES. Considering the duration of ES was 0.1 s, this delay appears reasonable compared to electromechanical delay times reported between the onset of electrical activity and measurable tension. Due to differences in experimental methods, this value has been reported to be between 0.01-0.120 s [26, 27], with 0.0495 s mean delay under eccentric activity and 0.0555 s mean delay under concentric activity [26]. Using this peak ES-induced change in wave speed, it was determined that wave speed was significantly different with ES than without ES when ES was applied near the end of swing phase for all amplitude conditions (Fig 11). The increased tendon tension observed with stimulation around this phase may have contributed to subjects ending swing phase sooner and decreasing their stride period. For the *Max* amplitude condition, wave speed was also different when ES was applied during early- to mid-stance (0-28% gait phase), and for the *Mid* amplitude condition only when ES was applied immediately after heel strike (0-7% gait phase).

It is noteworthy that electrical stimulation near the time of peak muscle activity in late stance did not lead to any change in Achilles tendon wave speed, an indicator of tendon tension. It appears that electrical stimulation on top of already-high muscle activity does not further increase muscle activation. This agrees with literature showing that addition of electrical stimulation simultaneously with voluntary exercise did not increase the training response in muscle strength over electrical stimulation or exercise alone [33], which suggested that electrical stimulation did not increase the contraction intensity. This is likely due to the fundamental differences between electrically and volitionally elicited contractions. Electrical stimulation recruits motor units in the reverse order of voluntary contractions, recruiting the larger type II fast-twitch motor units first [34]. Since these motor units are probably already active in the intense contraction of late stance, electrical stimulation likely did not lead to any further recruitment of motor units and thus did not lead to an increase in force. This finding does not support our hypothesis that the biomechanical effect of augmenting push-off would cause entrainment of the stimulus around that time, as the mechanical stimulus in Anklebot studies did [13, 14, 18]. In our case, there is no evidence that a mechanical stimulus was realized around the time of push-off. Nevertheless, late stance was the most common phase for entrained stride, and this fact leads to a question: if it is not to exploit augmented push-off, why does the body entrain to that specific phase? One potential reason is that the stimulus does create a gait disturbance (to stride period and Achilles tendon wave speed) at other times, and the easiest way to reject this is to align the stimulus to a time when this disturbance disappears. It is also possible that stimulation in late stance augmented push-off by increasing stride length and stride time without significantly affecting tendon tension. The stride period analysis showed an increase in stride period with stimulation in late stance (Fig 12), which would correspond to an increase in stride length as well for walking with constant speed on a treadmill.

One limitation of this study is that only women were tested. We don’t expect any different effects, but it should be investigated separately for sex differences. Another limitation is the small sample size for the overground walking and muscular response experiments. The muscle part was exploratory, using a new experimental technology: Achilles tendon tensiometry [24, 35]. The overground part was also exploratory, and will be expanded in follow-up studies. Another limitation is the possibility of muscle fatigue during the stimulation experiments. At the relatively low amplitudes used for the continual electrical stimulation (Experiments A and B) we do not believe muscle fatigue was an issue during normal walking. Experiment C only used intermittent electrical stimulation (each separated by 4-6 strides) so we do not believe muscle fatigue was an issue during that protocol either. Future studies using EMG data can be used to determine this. Finally, the cumulative effects of intermittent but persistent stimulation (Experiment C) on stride period are not known; any gradual drift in stride period that may have occurred in the non-ES strides is accounted for only by the standard deviation of non-ES stride period in Fig 12. With relatively sparse stimuli we did not expect such drift, but the effect could be studied in future research, for example using repeated bouts with and without intermittent stimulation. Similarly, it was assumed that ES only affected the stride it occurred in and the immediately following stride, but it is possible that each stimulus has effects that persist longer. Further research could seek to quantify such persistence and thereby determine the optimal experimental delay between stimuli.

Overall, the results of this study demonstrated that electrical stimulation of the gastrocnemius had an effect on gait that depended on the gait phase of stimulation. Rhythmic electrical stimulation entrained at phases that appear related to increasing stride period (around push-off) or decreasing stride period (around foot contact). The existence of these preferred phases could suggest that rhythmic electrical stimulation can be used to both increase and decrease cadence and speed. In addition, the finding of no change in Achilles tendon wave speed for stimulation in late stance could indicate a phase for electrical stimulation of the medial gastrocnemius that can lead to entrainment of gait without potentially negative gait disturbance effects. These findings suggest that electrical stimulation of the plantarflexor muscles could be useful for inducing changes in gait rhythm. Rhythmic electrical stimulation of the plantarflexors could lead to a new form of gait rehabilitation, for example to overcome gait disruptions in Parkinson’s disease or to promote rhythmic gait in stroke. A simple, noninvasive and unobtrusive electrode system would be a cost effective way to rehabilitate gait or manage it as an assistive device.

## Conclusions

We found that for both treadmill and overground walking, when the stimulation period was set to the subject’s stride period, entrainment was observed with phasing that either aligned the stimuli with ankle push-off or just before foot contact. This timing was likely related to the effect ES has on stride period when applied at that timing. Future studies using stimulation periods slightly greater and less than the natural stride period will investigate whether the relationship between stride period and stimulation period can explain the bimodal distribution of gait phase of stimulus during entrained strides.

We also found that a greater number of entrained strides were observed for treadmill walking than for overground walking. This was likely due to testing a stimulation period approximately equal to the stride period for perturbations that would be expected to alter subjects’ stride period. Future studies using stimulation periods different than stride period will investigate whether entrainment is still observed more frequently during treadmill walking than overground walking.

Measurements of wave speed along the Achilles tendon showed an effect related to when in the gait cycle ES was applied. The greatest effects were seen during early stance and late swing phases, with wave speed greater compared to wave speed without ES. Future studies will use EMG sensors and motion capture to determine the effect on multiple lower leg muscles and joints.

## Supporting information

S1 Raw data (ZIP)
